# Delivery of natural phenolic compounds for the potential treatment of lung cancer

**DOI:** 10.1007/s40199-019-00267-2

**Published:** 2019-05-21

**Authors:** Ashley G. Muller, Satyajit D. Sarker, Imran Y. Saleem, Gillian A. Hutcheon

**Affiliations:** School of Pharmacy & Biomolecular Sciences, Liverpool John Moores University, James Parsons Building, 3 Byrom Street, Liverpool, L3 3AF UK

**Keywords:** Phenolic compound, Lung cancer, Drug delivery, Polymeric nanoparticle, Pulmonary delivery

## Abstract

The application of natural products to treat various diseases, such as cancer, has been an important area of research for many years. Several phytochemicals have demonstrated anticarcinogenic activity to prevent or reduce the progression of cancer by modulating various cellular mechanisms. However, poor bioavailability has hindered clinical success and the incorporation of these drugs into efficient drug delivery systems would be beneficial. For lung cancer, local delivery via the pulmonary route would also be more effective. In this article, recent in vitro scientific literature on phenolic compounds with anticancer activity towards lung cancer cell lines is reviewed and nanoparticulate delivery is mentioned as a possible solution to the problem of bioavailability. The first part of the review will explore the different classes of natural phenolic compounds and discuss recent reports on their activity on lung cancer cells. Then, the problem of the poor bioavailability of phenolic compounds will be explored, followed by a summary of recent advances in improving the efficacy of these phenolic compounds using nanoparticulate drug delivery systems.

**Graphical abstract:**
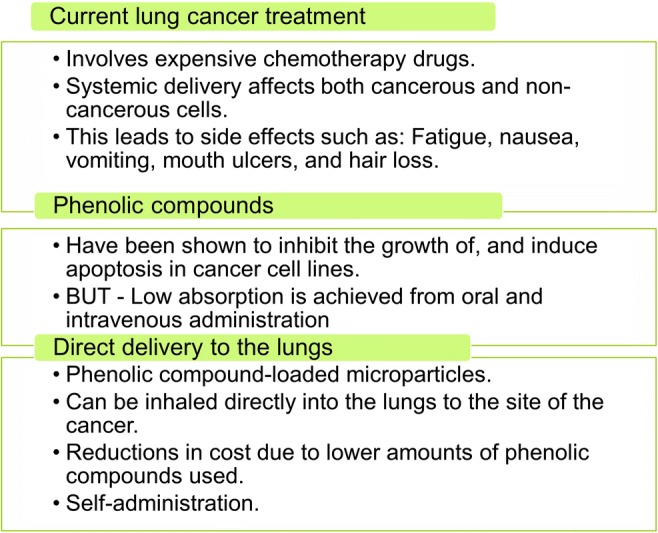
The rationale for direct delivery of phenolic compounds loaded in microparticles to the lungs

The rationale for direct delivery of phenolic compounds loaded in microparticles to the lungs

## Introduction

According to Cancer research UK, lung cancer is the third most common cancer in the UK with around 46,700 new cases reported each year, accounting for 21% of all cancer deaths and it has the second lowest ten-year survival rate (5%) of all cancers [1] . This is despite the fact that 89% of all lung cancers are linked to lifestyle or environmental risk factors and hence preventable [1]. Cancer chemoprevention consist of the chronic use of a synthetic, natural or biological agent to reduce or inhibit the formation and progression of cancer, in which oxidative stress is a key factor [2]. Plants and fungi produce secondary metabolites, such as phenolic compounds, as a protective measure against oxidative stress caused by ultraviolet light, insects, viruses, and bacteria [3]. Therefore, it is postulated that these same metabolites could be applied to help protect humans from diseases, such as cancer, caused by oxidative stress. A review on the link of oxidative stress in cancer is beyond the scope of this review, but can be found in several sources [4, 5]. It should be noted that current literature is divided as to whether or not phenolic compounds used in conjunction with chemotherapy and/or radiation therapy reduces the efficacy of cancer treatment [6–8]. It is the intention that the phenolic compounds discussed in this review be considered for use as a treatment for lung cancer, in and of itself, and not to be used in conjunction with current cancer chemotherapy and/or radiation treatment. Several different techniques are used to extract, separate, and identify phenolic compounds from natural sources in a pure form required for clinical use, including liquid-liquid extraction, solid-liquid extraction, supercritical fluid extraction, high performance liquid chromatography, supercritical fluid chromatography, mass spectrometry, nuclear magnetic resonance spectroscopy, amongst others [9, 10].

A previous systematic review discovered that eating fruits and vegetables can confer up to an 18% decrease in the risk of developing lung cancer [11]. It is hypothesised that the reason behind the decreased lung cancer risk is due to the high amounts of flavonoids, and other phenolic compounds, present in the fruits and vegetables [12, 13].

The following section will list and describe the categories of the different phenolic compounds and examples of them that have been shown to have anticancer properties.

## Phenolic compounds

Phenolic compounds are diverse in structure, but are identified as having at least one aromatic ring possessing one or more hydroxyl groups [14]. Several classes of phenolic compounds exist, namely, flavonoids, phenolic acids, phenolic alcohols, stilbenes and lignans [10, 15]. Phenolic compounds are ubiquitous, being present in almost all of the foods we consume, from plant derived foods, including fruits, vegetables, legumes and cereals to beverages such as beer, coffee, tea, wine and also in spices and herbs, such as cinnamon, curcumin, sage, and thyme [16–19].

### Flavonoids

**Fig. 1:**
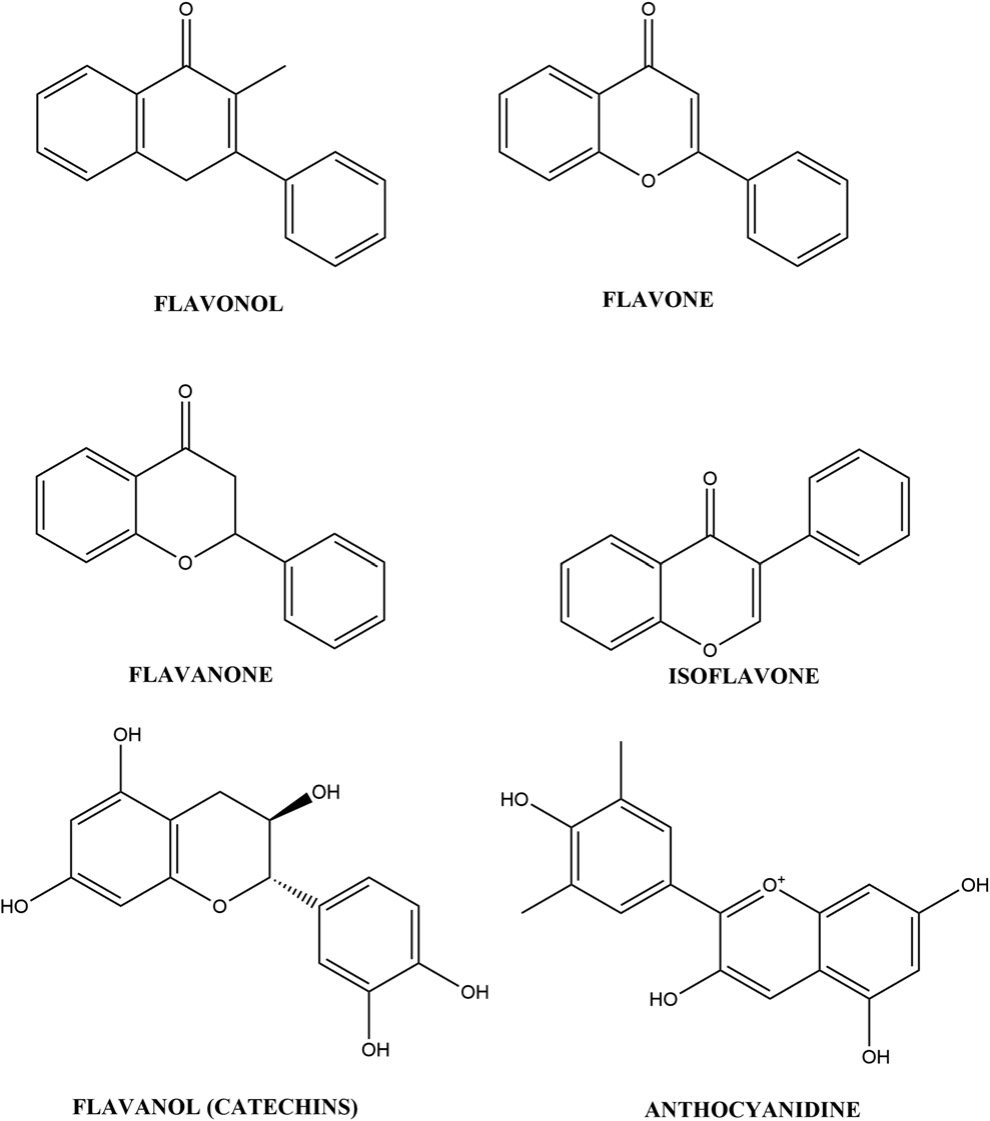
Chemical structure of flavonoids

**Fig. 2:**
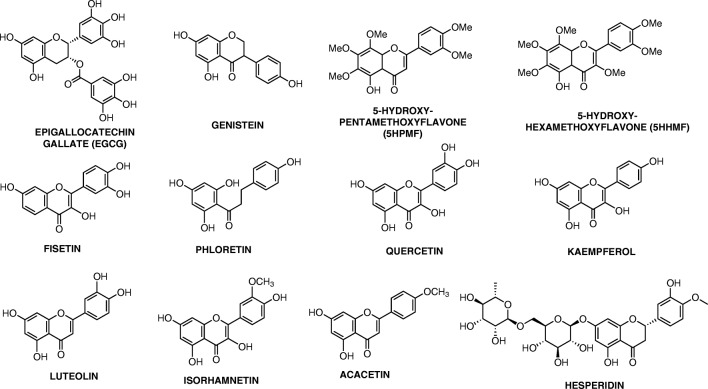
Chemical structures of flavonoids listed in paper

Flavonoids, the largest and most widely studied class of phenolic compounds, can be subdivided into flavonols, flavones, flavanones, isoflavones, anthocyanidins, and catechins [10]. Tannins are flavonoids that, as a result of the plants themselves or from food processing, are polymerised into large molecules [20]. There are two types of tannins, namely hydrolysable tannins and condensed tannins. Hydrolysable tannins (which contain glucose or another polyol as their central core) are subclassed as gallotannins (core esterification by gallic acid) or ellagitannins (core esterification by hexahydroxydiphenic acid) [21]. Condensed tannins are also known as proanthocyanidins and are polymeric or oligomeric compounds made from flavan-3-ol [21]. The chemical structures of the main classes of flavonoids and the examples listed below are presented in Figs. 1 and 2, respectively. Refer to Table 1 for the list of studies discussed below.

**Table 1:** List of flavonoids with potential anticancer activity against various lung cancer cell lines

Flavonoid	Concentration	Cell line	Mechanisms	Reference
EGCG	20 μM	A549 H1650 H460	Upregulation of TP53 causing growth inhibition	[29]
	5–50 μM	H1299 H460	Increased expression of miR-210, leading to growth inhibition	[30]
	70 μM	H69 H69VP	Reduced tolomerase activity, apoptosis induction, DNA fragmentation, and cell cycle arrest	[31]
	2.5–40 μmol/L	H2122 H358 H460 H1975 H1993	Inhibition of cell proliferation	[32]
5HPMF	16.5 μM	H1299	Apoptosis through activation of caspase-3	[34]
5HHMF	16.5 μM	H1299	Apoptosis through activation of caspase-3	[34]
Genistein	10 μM + 50 ng/mL trichostatin A	A549	Enhanced inhibition of growth and increased apoptosis by increasing TNFR-1 death receptor signalling	[35]
	25 μM	H3255 H1650 H1781	Decreased DNA-binding activity of NF-κB and a reduction in COX-2, pAkt, EGFR and PGE_2_ expression	[37]
	20–40 μM	SPC-A-1	Cell-cycle arrest, antiproliferation, induction of apoptosis via regulation of genes related to apoptosis	[38]
	15–30 μmol/L	H460	Nullified the NF-κB-inducing activity of cisplatin, docetaxel and doxorubicin increasing cell-growth inhibition and inducing of apoptosis	[39]
Fisetin	5–20 μM	A549	Inhibition of both PI3K/Akt and mTOR signalling through attenuating PI3K protein expression, inhibiting Akt and mTOR phosphorylation	[41]
	1, 5,10 μM	A549	Downregulation of ERK1/2, MMP-2, and u-PA. Inhibition of NF-κB and AP-1 binding. Decrease in the nuclear levels of NF-κB, c-Fos, and c-Jun	[42]
Phloretin	125–150 μg/mL	A549 Calu-1 H838 H520	Decreased proliferation, induction of apoptosis, Bcl-2 expression suppression, increased cleaved-caspase-3 and -9 protein expression, MMP-2 and -9 downregulation	[43]
	25, 50, 100 and 200 μM	A549	Inhibit migration, increase apoptosis via upregulating ERK, JNK, Bax and P38 MAPK and activating caspase-3 and -9, and TP53 while downregulating Bcl-2 and NF-κB	[44]
Quercetin	0.74–4.40 μmol/L	A549	Dose-dependent decrease in cell growth and an increase in apoptosis	[45]
Kaempferol	10–140 μM	A549	Dose-dependent antiproliferative activity and impaired metastasis via suppression of EMT	[56]
	25 μM	A549	EMT suppression induced by inhibiting the phosphorylation of Smad3 at Threonine-179 by Akt1	[57]
	30, 50 and 80 μM	H460	Apoptosis via induction caspase-3, AIF, and increasing antioxidant enzymes	[58]
Luteolin	20–80 μM	A549	Cell cycle arrest and inducing apoptosis through activating JNK, increasing Bax, promoting procaspase-9 cleavage, and activating caspase-3	[51]
	25–100 μM	A549	Apoptotic effect and reduction of cell motility and cell migration. Upregulation of caspase-3 and caspase-9, downregulation of Bcl-2, increase in expression of Bax, phosphorylation of mitogen-activated protein kinase and extracellular regulated protein kinase (MEK), and activation of Akt	[52]
	10–100 μM	A549 H460	Inhibition of cell proliferation and increased apoptosis via upregulation of a microRNA (miR-34a-5p) that targets an oncogene (MDM4)	[53]
	20–80 μM	A549 H460	Decrease in cell proliferation by downregulation of the Tyro3, Axl, MerTK (TAM) receptor tyrosine kinases (RTK)	[54]
	20–160 μM	H460	Antiproliferative effects via Sirt1-mediated apoptosis	[55]
Isorhamnetin	16 μM	A549	Inhibition of cellular proliferation and colony formation and an increase in apoptosis via the mitochondria-dependent pathway with caspase activation	[47]
	25 μM	A549	Synergistically increase the antiproliferative and proapoptotic effects of the anticancer drugs via disruption of the mitochondrial membrane potential and activation of caspases and PARP	[48]
Hesperidin	5–50 μM	A549 NCIH358	Inhibition of proliferation and induction of apoptosis via loss of mitochondrial membrane potential, activation of caspase-3, and affecting the fibroblast growth factor and NF-κB signal transduction pathways	[60]
	5–100 μM	H1993	Suppression of cell viability	[61]
Acacetin	1–30 μM	A549	Inhibition of cell viability, invasion and migration via disruption of several signalling pathways and kinases including AP-1, NF-κB, c-Fos, c-Jun, MLK3, MAPK3/6, and p38a MAPK	[62]

Epigallocatechin 3-gallate (EGCG) is the most abundant catechin (flavonoid) present in both black- and green tea (*Camellia sinensis*) (refer to Fig. 2 for structure). It has been shown to have several actions, inter alia, anti-oxidative [22], anti-inflammatory [23], anticancer [24], promotion of cell cycle arrest [25], inhibition of cellular proliferation [26], proapoptotic [27], antimetastatic and anti-angiogenic [28]. The antitumour activity of EGCG is postulated to be due to its interaction with several signalling pathways. The pathways include protein kinase suppression, inhibition of transcription factors such as nuclear factor kappa-light-chain-enhancer of activated B cells (NF-κB), epidermal growth factor receptor (EGFR), activator protein-1 (AP-1) and signal transducer and activator of transcription proteins (STATs), and mechanisms such as induction of apoptosis or cell cycle arrest and prevention of metastasis [29–32]. A study by Jin et al. [29] found that treating three human lung cancer cell lines (A549, H1650 and H460) with 20 μM EGCG inhibited anchorage-independent growth of all three cell lines via upregulation of p53 expression, increased phosphorylation of tumour protein p53 (TP53) at anti-phospho-p53 (Ser^15^) and anti-phospho-p53 (Ser^20^) and enhancement of its transcriptional activity, as well as inhibition of mouse double minute 2 (MDM2)-mediated TP53 ubiquitination. Another study found that treatment of EGCG (5–50 μM) resulted in increased expression of miR-210, leading to growth inhibition of human non-small cell lung cancer cell lines, H1299 and H460 [30]. The effect of EGCG on drug-sensitive (H69) and drug-resistant (H69VP) small-cell lung carcinoma (SCLC) cells was studied. It was found that exposure of both cell lines to 70 μM EGCG for 24 h resulted in a 50–60% reduction in telomerase activity with initiation of apoptosis through decreased activity of caspases-3 and -9, DNA fragmentation in cells, and cell-cycle arrest [31]. A similar study assessed the effect of EGCG on various human non-small cell lung cancer (NSCLC) cell lines (H2122, H358, H460, H1975, and H1993) that were either erlotinib-sensitive, erlotinib-resistant, showed c-Met overexpression and/or had acquired erlotinib resistance. Exposure of the cell lines to 2.5–40 μmol/L of EGCG resulted in a dose-dependent inhibition of cell proliferation [32]. EGCG co-administered with the anti-lung cancer drug, leptomycin, showed a synergistic increase in cytotoxicity of the human lung cancer A549 cells [33].

Two flavonoids, 5-hydroxy-3,7,8,3′,4′-pentamethoxyflavone (5HPMF), and 5-hydroxy-3,6,7,8,3′,4′-hexamethoxyflavone (5HHMF), are found in sweet orange (*Citrus sinensis*) (refer to Fig. 2 for structures). These two flavonoids were shown to initiate apoptosis through activation of caspase-3 and cleavage of poly(ADP-ribose) polymerase (PARP) (a substrate of activated caspase-3) as well as downregulating oncogenic proteins, such as inducible nitric oxide (iNOS), cyclooxygenase (COX-2), myeloid cell leukemia-1 (Mcl-1), and K-ras in human lung carcinoma H1299 cells [34]. The inhibitory concentration (IC_50_) values for the two flavonoids after 24 h was recorded as 16.5 μM.

Genistein, also known as 5,7-dihydroxy-3-(4-hydroxyphenyl)-4H-1-benzopyran-4-one and 4′,5,7-trihydroxyisoflavone, is the most abundant isoflavone found in soybean (*Glycine max*) (refer to Fig. 2 for structure). Shiau et al. [35] exposed A549 cells to a combination of 10 μM genistein and 50 ng/mL of trichostatin A (TSA), resulting in enhanced inhibition of growth and increased apoptosis, thought partly to be due to increased caspase-3 activity. A subsequent study revealed that the same combination augmented the anticancer effect of TSA by increasing tumour necrosis factor (TNF) receptor-1 (TNFR-1) death receptor signalling [36]. Gadgeel et al. [37] studied the effect of genistein in combination with epidermal growth factor receptor tyrosine kinase inhibitors (EGFR-TKIs), erlotinib and gefitinib on NSCLC cell lines with various EGFR mutations and sensitivities to EGFR-TKIs, H3255, H1650, and H1781 (wild-type EGFR). Genistein (25 μM) in combination with erlotinib/gefitinib increased the growth inhibition and apoptosis in all three cell lines postulated to be due to decreased DNA-binding activity of NF-κB and a reduction in COX-2, pAkt, EGFR and prostaglandin E2 (PGE_2_) expression [37]. Exposure of the human lung adenocarcinoma SPC-A-1 cell line to 20–40 μM genistein resulted in cell-cycle arrest, antiproliferation and induction of apoptosis via regulation of genes related to apoptosis, especially genes from the B cell lymphoma 2 (Bcl-2) family and TNF ligand and receptor family [38]. Treatment of H460 cells with 15–30 μmol/L genistein combined with cisplatin, docetaxel or doxorubicin resulted in a greater synergistic effect cell-growth inhibition and induction of apoptosis than compared with either one by itself [39]. It was found that the pre-exposure of the cells to the genistein inactivated NF-κB thereby nullifying the NF-κB-inducing activity of cisplatin, docetaxel and doxorubicin [39].

The flavonoid, fisetin (3,7,3′,4′-tetrahydroxyflavone) is naturally found in several foods including grape, persimmon, strawberry apple, onion, and cucumber [40] (refer to Fig. 2 for structure). Khan et al. [41] exposed A549 cells to 5–20 μM fisetin causing a dose-dependent inhibition of both phosphoinositide 3-Kinase/protein kinase B (PI3K/Akt) and mammalian target of rapamycin (mTOR) signaling through attenuating PI3K protein expression, inhibiting Akt and mTOR phosphorylation. Fisetin (1, 5 and 10 μM) was shown to inhibit the ability of A549 cells to adhere, migrate, and invade, by interfering with the regulation of extracellular signal-regulated kinase 1 and 2 (ERK1/2), matrix metalloproteinase-2 (MMP-2), and urokinase-type plasminogen activator (u-PA) at both the protein and microRNA (miRNA) levels [42]. There was also a concentration-dependent inhibitory effect on NF-κB and AP-1 binding with a significant decrease in the nuclear levels of NF-κB, c-Fos, and c-Jun [42].

Phloretin, 3-(4-hydroxyphenyl)-1-(2,4,5-trihydroxyphenyl), is a flavonoid from several sources including apples and plants, such as *Hoveniae Lignum, Pieris japonica, and Loiseleuria procumbens* [43] (refer to Fig. 2 for structure). It was found that administration of 125–150 μg/mL of phloretin to NSCLC cell lines A549, Calu-1, H838 and H520 caused a dose-dependent decrease in proliferation and induction of apoptosis through suppressing the expression of Bcl-2, increasing cleaved-caspase-3 and -9 protein expression, and downregulating MMP-2 and -9 expression on gene and protein levels [43]. Min et al. [44] showed that phloretin (25, 50, 100 and 200 μM) caused a dose- and time-dependent inhibition of migration and an increase in apoptosis of A549 cells through upregulating ERK, c-Jun N-terminal kinases (JNK), Bcl-2-associated X protein (Bax) and P38 mitogen-activated protein kinases (MAPK) and activating caspase-3 and -9, and TP53 while downregulating Bcl-2 and NF-κB.

Quercetin (3,3′,4′,5,7-pentahydroxyflavone) is the most common flavonol distributed in various plants and plant foods (refer to Fig. 2 for structure). Zheng et al. [45] studied the effect of quercetin (0.74–4.40 μmol/L) administration on A549 cells. It was found that quercetin caused a dose-dependent decrease in cell growth and an increase in apoptosis.

Isorhamnetin is a flavonoid that is an immediate metabolite of quercetin in mammals [46] (refer to Fig. 2 for structure). Ruan, Hu and Chen [47] showed that administration of 16 μM isorhamnetin to A549 cells resulted in inhibition of cellular proliferation and colony formation and an increase in apoptosis via the mitochondria-dependent pathway with caspase activation. Isorhamnetin (25 μM) when combined with 0.5 μM each of cisplatin and carboplatin, synergistically increased the antiproliferative and proapoptotic effects of these anticancer drugs in A549 cells via disruption of the mitochondrial membrane potential and activation of caspases and PARP [48].

Luteolin, 3′,4′,5,7-tetrahydroxyflavone, is a flavone found naturally in its glycosylated form in various green vegetables including artichoke, broccoli, cabbage, celery, cauliflower, green pepper, and spinach [49, 50] (refer to Fig. 2 for structure). Administration of 20–80 μM luteolin to A549 lung cancer cells caused a dose- and time-dependent cytotoxic effect by causing cell cycle arrest and inducing apoptosis through activating JNK, increasing Bax, promoting procaspase-9 cleavage, and activating caspase-3 [51]. Meng et al. [52] showed that luteolin (25–100 μM) had a dose- and time-dependent antiproliferative and apoptotic effect on A549 lung cancer cells, also significantly reducing cell motility and cell migration. Luteolin was shown to upregulate caspase-3 and caspase-9, downregulate Bcl-2, increase expression of bax, phosphorylate mitogen-activated protein kinase and ERK (MEK), and activate Akt [52]. Jiang et al. [53] caused a dose- and time-dependent inhibition of cell proliferation and increased apoptosis when administering luteolin (10–100 μM) to human lung cancer A549 and H460 cells. The mechanism of action was found to be the upregulation of a microRNA (miR-34a-5p) that targets an oncogene (MDM4) [53]. Luteolin (20–80 μM) caused a decrease in cell proliferation by downregulation of Tyro3, Axl, MerTK (TAM) receptor tyrosine kinases (RTK) in parental and cisplatin-resistant human lung cancer A549 and H460 cells [54]. Ma et al. [55] showed that luteolin (20–160 μM) caused antiproliferative effects in human lung cancer NCI-H460 cells through Sirt1-mediated apoptosis.

Kaempferol (3, 4′,5,7-tetrahydroxyflavone) is another common dietary flavonoid (refer to Fig. 2 for structure). Hang et al. [56] administered 10–140 μM kaempferol to A549 cells and show that it had dose-dependent antiproliferative activity, with an IC_50_ value of 72 μM after 24 h of incubation, and impaired metastasis of the cells via suppression of Epithelial-Mesenchymal Transition (EMT). Another study pretreated A549 cells with 25 μM kaempferol and found the EMT suppression induced by kaempferol was a result of inhibition of the phosphorylation of Smad3 at Threonine-179 by Akt1 [57]. Exposure of H460 cells to 30, 50 and 80 μM kaempferol resulted in a dose-dependent increase in apoptosis via induction of caspase-3, apoptosis-inducing factor (AIF) and increasing antioxidant enzymes [58].

Hesperidin ((2*S*)-5-hydroxy-2-(3-hydroxy-4-methoxyphenyl)-7-[(2*S*,3*R*,4*S*,5*S*,6*R*)-3,4,5-trihydroxy-6-[[(2*R*,3*R*,4*R*,5*R*,6*S*)-3,4,5-trihydroxy-6-methyloxan-2-yl]oxymethyl]oxan-2-yl]oxy-2,3-dihydrochromen-4-one) is a flavanone that is found in many citrus fruits [59] (refer to Fig. 2 for structure). A study by Cincin et al. [60] found that 5–50 μM hesperidin caused a dose- and time-dependent inhibition of proliferation and induction of apoptosis via loss of mitochondrial membrane potential, activation of caspase-3, and affecting the fibroblast growth factor and NF-κB signal transduction pathways in A549 and NCI-H358 cells. Hesperidin (5–100 μM) showed a significant inhibitory effect on tyrosine kinase inhibitors (TKI)- resistant cell line, H1993, while almost having no effect on the TKI- sensitive cell line, H2073 [61]. The study did not speculate on the method of this inhibitory effect.

Acacetin (5,7-dihydroxy-40 -methoxyflavone) is a flavonoid that has been studied for its effect on lung cancer cells (refer to Fig. 2 for structure). Chien et al. [62] administered 10–30 μM acacetin to A549 which resulted in significant inhibition of cell viability. Further exposure of the A549 cells to 0, 1, 2.5, and 5 μM acacetin showed an inhibition of invasion and migration thought to be due to disruption of several signalling pathways and kinases including AP-1, NF-κB, c-Fos, c-Jun, mixed lineage protein kinase 3 (MLK3), mitogen-activated protein kinases 3/6 (MAPK3/6), and p38a MAPK.

### Phenolic acids

**Fig. 3:**
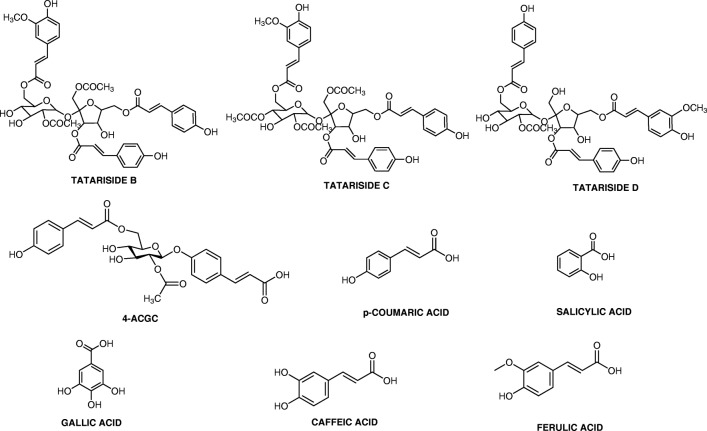
List of phenolic acids and their chemical structures

Phenolic acids can be subdivided into two major subgroups, namely hydroxybenzoic acids and hydroxycinnamic acids [3]. Refer to Fig. 3 for the structures of phenolic compounds discussed below and refer to Table 2 for the list of studies discussed below.

**Table 2:** List of phenolic acids

Phenolic acid name	Concentration	Cell line	Mechanisms	Reference
Tatariside B Tatariside C Tatariside D	18.3 μg/mL 6.44 μg/mL 2.83 μg/mL	A549	Inhibition of proliferation of cell line	[63]
4-ACGC	37.73 μg/mL 50.6 μg/mL 62.0 μg/mL	A549 NCI-H1299 HCC827	Upregulation of caspase-3 & 9, Bad, and Bax down-regulation of Bcl-2	[64]
p-Coumaric acid	50–100 μM	H1993	Suppression of cell viability	[61]
	50–1000 μM	A549	Decreased proliferation, superoxide anion production, cell adhesion, and cell migration	[66]
Salicylic acid	6.0 mM	A549	Proapoptotic, antiproliferative, and cytotoxic effects	[68]
Gallic acid	10–50 μM 100–200 μM	Calu 6 A549	Depletion of glutathione and increasing ROS levels	[70]
	50 μM	H1975	Inhibition of Src-mediated STAT3 phosphorylation, leading to downregulation of STAT3 target genes (Bcl2 and cyclin D) causing apoptosis and cell cycle arrest	[71]
	5 μM	H1993	Inhibition of Src-mediated STAT3 phosphorylation, leading to downregulation of STAT3 target genes (Bcl2 and cyclin D) causing apoptosis and cell cycle arrest	[61]
Caffeic acid	50–1000 μM	A549	Decreased proliferation, superoxide anion production, cell adhesion, and cell migration	[66]
Ferulic acid	50–1000 μM	A549	Decreased proliferation, superoxide anion production, cell adhesion, and cell migration	[66]

The hydroxycinnamic acid derivatives, tatariside B, C and D, are isolated from tartary buckwheat (*Fagopyrum tataricum*) [63]. The tatarisides B-D showed significant cytotoxicity effects against human lung adenocarcinoma A-549 cells, with recorded IC_50_ values of 18.3 μg/mL, 6.44 μg/mL, and 2.83 μg/mL, respectively [63].

A study of the effect of 4-O-(2″-O-acetyl-6″-O-p-coumaroyl-β-d-glucopyranosyl)-p-coumaric acid (4-ACGC) against several lung cancer cell lines, including A549, NCI-H1299, and HCC827 showed that 4-ACGC caused dose-dependent, anti-proliferative activity between 10 and 100 μg/mL, with IC_50_ values of 37.73 μg/mL (A549), 50.6 μg/mL (NCI-H1299), and 62.0 μg/mL (HCC827) [64]. This suggests that 4-ACGC causes upregulation of caspase-3 & 9, Bcl-2-associated death promoter (Bad), and Bax while also down-regulating Bcl-2 [64].

p-Coumaric acid (4-hydroxycinnamic acid) is biologically synthesised through the shikimate pathway with phenylalanine and tyrosine as precursors [65]. p-Coumaric acid (50–100 μM) showed a significant inhibitory effect on the proliferation of the TKI-resistant cell line, H1993, while only moderately affecting the TKI- sensitive cell line, H2073 [61]. The study did not speculate on the method of this inhibitory effect. Nasr Bouzaiene et al. [66] found that p-Coumaric acid (50–1000 μM) caused up to a 55% reduction in the proliferation of A549 cells in a dose-dependent manner. p-Coumaric acid (50–200 μM) also caused a significant decrease in the production of superoxide anion, cell adhesion, and tumour cell migration in A549 cells in a dose-dependent manner [66].

Salicylic acid (2-Hydroxybenzoic acid) is a phenolic acid that was first isolated from white willow (*Salix alba*) and has demonstrated anti-inflammatory properties [67]. Vejselova and Kutlu [68] discovered that salicylic acid had proapoptotic, antiproliferative, and cytotoxic effects on A549 cells with a recorded IC_50_ of 6.0 mM after 24 h.

Gallic acid (3,4,5-trihydroxybenzoic acid) is a phenolic acid from various sources such as green tea, raspberries, blueberries, bananas, and grapes [69]. Gallic acid has been shown to inhibit cell growth and induce cell death in Calu 6 (IC_50_ 10–50 μM) and A549 (IC_50_ 100–200 μM) cells by depleting glutathione and increasing reactive oxygen species (ROS) levels [70]. Gallic acid was also found to have an anti-proliferative effect on TKI-resistant cell line, H1975, at 50 μM while not affecting TKI- sensitive cell lines [71]. Another study also found that gallic acid (5 μM) showed a strong inhibitory effect on the TKI- resistant cell line, H1993, while sparing the TKI- sensitive cell lines [61]. Both studies suggest that gallic acid inhibits TKI-resistant cell line proliferation through inhibition of Src-mediated signal transducer and activator of transcription protein 3 (STAT3) phosphorylation, leading to downregulation of STAT3 target genes (Bcl2 and cyclin D) causing apoptosis and cell cycle arrest [61, 71].

Caffeic acid (50–1000 μM) caused a significant reduction in A549 cell viability in a dose-dependent manner [66]. Caffeic acid (50–200 μM) also caused a significant decrease in the production of superoxide anion, cell adhesion, and tumour cell migration in A549 cells in a dose-dependent manner [66].

Ferulic acid (50–1000 μM) caused a significant reduction in A549 cell viability in a dose-dependent manner [66]. Ferulic acid (50–200 μM) also caused a significant decrease in the production of superoxide anion, cell adhesion, and tumour cell migration in A549 cells in a dose-dependent manner [66].

### Diphenylalkaloids

**Fig. 4:**
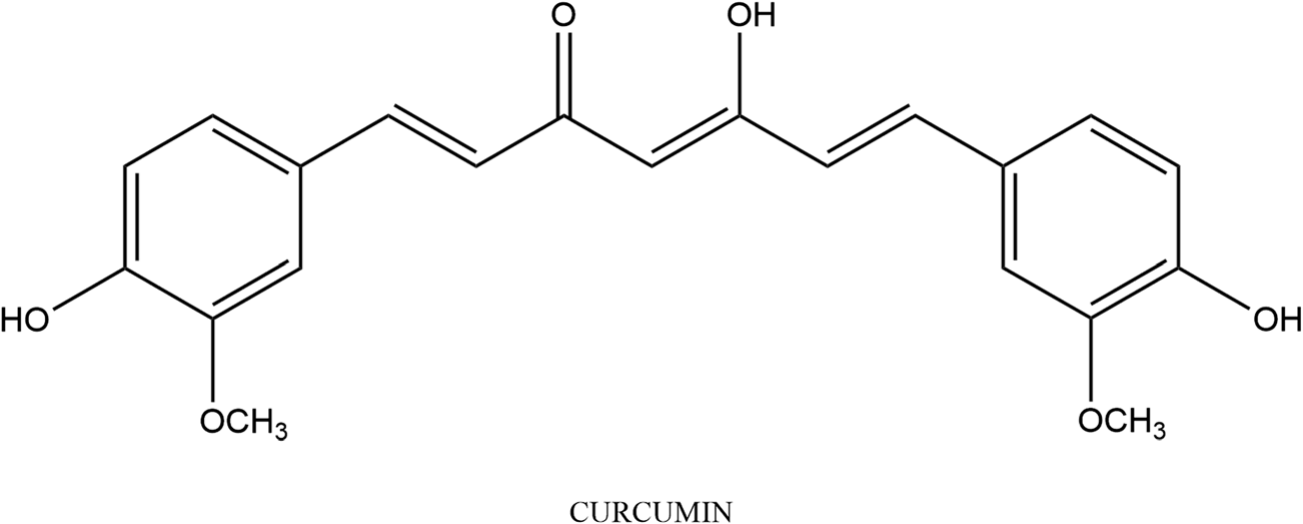
Curcumin structure

Diphenylalkaloids are alkaloids with one or more diphenyl ether linkages [72]. Diphenylalkaloids can be further classified, depending on the length of the carbon chain between two aromatic rings, into diphenylheptanoids, diphenylpentanoids, and other diphenylalkanoids [73]. Refer to Fig. 4 for structure and Table 3 for the list of studies discussed below.

**Table 3:** List of studies where curcumin showed anticancer activity against various lung cancer cell lines

Diphenylalkaloid	Concentration	Cell line	Mechanisms	Reference
Curcumin	30 μM	A-549	Caspase-3 induced apoptosis. TP53-independent apoptosis	[81]
	40–50, 160 μM	A549 H1299	TP53-independent induction of apoptosis	[82]
	10–20 μM	A549	MMP 2&9 mediated inhibition of invasion and metastasis	[83]
	30 μM	NCI-H460	Caspase-3 & 8 induced apoptosis	[84]
	5–20 μmol/L	CL1–5	Upregulation of tumour suppressor HLJ1	[85]
	50 μM	PC-9	Apoptosis via upregulation of GADD 45 and 153	[86]
	15 μmol/L	SK-MES-1	Upregulation and downregulation of genes	[87]

Curcumin ((1*E*,6*E*)-1,7-Bis(4-hydroxy-3-methoxyphenyl)-1,6-heptadiene-3,5-dione) consists of two groups of diphenylalkaloids, namely diphenylheptanoids (or diarylheptanoids) and diphenylpentanoids (or diarylpentanoids) [74, 75]. Curcumin is a hydrophobic polyphenol responsible for the yellow colour of the Indian spice turmeric (*Curcuma longa)* [76]. Curcumin is considered the most active constituent of turmeric comprising 2–5% of turmeric preparations. Turmeric has been used for over 5000 years in the traditional Indian medicine system known as Ayurveda [77]. Recent evidence suggests that curcumin has both antioxidant and anti-inflammatory properties [78, 79].

Curcumin has been shown to modulate cytokines, enzymes, growth factors, kinases, and transcription factors [80]. Several researchers investigated the anticancer properties of curcumin. A study by Lin et al. [81] found that curcumin at a concentration of 30 μM activated caspase-3 resulting in DNA damage and endoplasmic reticulum (ER) stress and mitochondrial-dependent-induced apoptosis in human lung cancer A-549 cells. The effect of curcumin was assessed on two human lung cancer cell lines, namely A549 (TP53 proficient) and the large cell lung carcinoma cell line H1299 (TP53 null mutant) [82]. Curcumin inhibited the growth and induced apoptosis in a concentration dependent manner in both cell lines. Exposure of the cell lines to 40–50 μM resulted in a 50% reduction in cell viability, while a concentration of 160 μM lead to a more significant 95% reduction in the viability of the cells. Since curcumin induced apoptosis occurred in both the TP53 proficient (A549) and the TP53 deficient (H1299) cell line it can be deduced that curcumin induces its growth inhibitory effect in a TP53-independent manner. Curcumin was also shown to inhibit the invasion and migration of A549 cells through the inhibition of MMP-2 and matrix metalloproteinase-9 (MMP-9) and Vascular Endothelial Growth Factor (VEGF) at concentrations of 10 and 20 μM [83]. Wu et al. [84] studied the effects of curcumin on human non-small cell lung cancer NCI-H460 cells. They found that curcumin had a dose-dependent cytotoxic effect on the NCI-H460 cells with a concentration of 30 μM leading to cell death in 95% of the cells. The study ascertained that curcumin caused apoptosis in the NCI-H460 cells due to mitochondrial membrane potential loss and subsequent caspase-3 activation, together with the activation of FAS/caspase-8 (extrinsic) pathway, ER stress proteins, growth arrest- and DNA damage-inducible gene 153 (GADD 153) and glucose-regulated protein 78 (GRP78). Curcumin (5–20 μmol/L) was also shown to concentration-dependently inhibit human lung adenocarcinoma cells (CL1–5) by invasion and metastasis via the upregulation of the DnaJ-like heat shock protein 40 (HLJ1) associated with tumour suppression, via activation of the JNK/JunD pathway [85]. It was also shown that curcumin caused significant growth inhibition of the human lung cancer cell PC-9, inducing G1 and S phase arrests in cell-cycle regulation and apoptosis in a TP53-independent manner [86]. Growth arrest and apoptosis was most significantly observed at a concentration of 50 μM, where the percentage of viable cells 24 h after treatment was 47.5% of the control. It was found that the apoptosis was driven by the upregulation of growth arrest- and DNA damage-inducible gene 45 (GADD 45) and GADD 153. Similarly, curcumin was shown to induce apoptosis in human lung squamous cell carcinoma (SK-MES-1) via upregulation of several genes including proliferating cell nuclear antigen (PCNA), DNA polymerase lambda (POLL), MutY DNA Glycosylase (MUTYH), signal transducer and activator of transcription 5a (STAT5A), and AKT1, and the downregulation of mitogen-activated protein kinase 1 (MAPK1), arrestin beta 2 (ARRB2), protein tyrosine kinase 2 (PTK2), mitogen-activated protein kinase 14 (MAPK14), vascular endothelial growth factor A (VEGFA), and nuclear factor kappa b subunit 1 (NFKB1); the most significant which was found at 15 μmol/L [87].

### Stilbenes

**Fig. 5:**
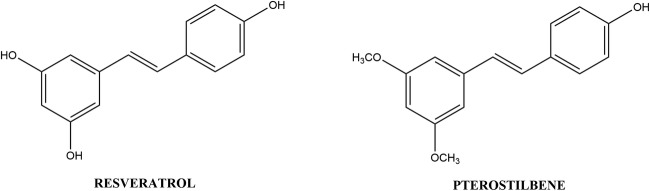
List of stilbenes and their chemical structures

Stilbenes are phenolic compounds with a core C_6_-C_2_-C_6_ structural feature. They are phytoalexins usually produced by plants in response to fungal, bacterial or viral attacks [88]. Refer to Fig. 5 for the structures of the stilbenes and Table 4 for the list of studies discussed below.

**Table 4:** List of stilbenes with potential anticancer activity against various cell cancer lines

Stilbenes name	Concentration	Cell lines	Mechanisms	References
Resveratrol	20 μM	A549	Suppressed invasion and metastasis by preventing TGF-β1 induced EMT	[93]
	4–64 μM	A549	Inhibition of growth and apoptosis induced via caspase 3 activation	[94]
	1–10 μM	H1993	Suppression of cell viability	[61]
Pterostilbene	10–100 μM	NCI-H460 SK-MES-1	Decrease in cell viability and increased apoptosis and caspase-3 & 7 activity	[96]

Resveratrol (*trans*-3,5,4′-trihydroxystilbene) is the most common natural stilbene found abundantly in a large number of fruits and vegetables, most notably grapes [89]. It has anti-inflammatory [90], anti-oxidative [91], proapoptotic and cell cycle arrest [92] properties. A study by Wang et al. [93] found that 20 μM resveratrol suppressed invasion and metastasis of A549 cells by preventing transforming growth factor beta 1 (TGF-β1) induced EMT. Another study exposed A549 cells to 4–64 μM resveratrol which resulted in inhibition of growth (IC_50_ 8.9 μM) and apoptosis induced via caspase-3 activation [94]. Resveratrol (1–10 μM) showed a significant inhibitory effect on TKI- resistant cell line, H1993, while almost having no effect on the TKI- sensitive cell line, H2073 [61]. The study did not speculate on the method of this inhibitory effect.

**Table 5:** List of phenolic compounds and the polymeric nanoparticles used in their delivery

Nanoparticulate system	Phenolic compound	Effect	Reference
PLGA PLGA-PEG	Curcumin	PLGA and PLGA-PEG nanoparticles: - increased the peak concentration of curcumin by 2.9- and 7.4-fold - increased the half-life of the curcumin from 1 h to 4 h (PLGA) and 6 h (PLGA-PEG) - enhanced the oral bioavailability of curcumin by 15.6- and 55.4-fold, respectively	[123]
Chitosan Gelatin Hyaluronan	Curcumin	Polymeric chitosan, gelatin, and hyaluronan nanoparticles: - All showed enhanced apoptotic effects of 45, 40 and 32%, respectively, as opposed to pure curcumin (>20%) on A549 cells	[124]
Chitosan	naringenin	naringenin encapsulated chitosan nanoparticles (NAR/CS NPs): - caused a statistically significant dose-dependent decrease in cell viability in A549 cells as compared with free naringenin, without affecting the normal 3T3 cells	[126]
Gelatin	Resveratrol	Resveratrol encapsulated in gelatin nanoparticles: - induced cell death in human lung cancer cells NCI-H460 by changing the expression of TP53, p21, caspase-3, Bax, Bcl-2 and NF-κB	[119]
Gelatin	Resveratrol	Resveratrol encapsulated gelatin nanoparticles (R-GNPs): - improved cellular uptake and superior bioavailability, decreasing cell viability, mitochondrial membrane potential and increasing cytotoxicity, DNA damage and intracellular ROS levels as compared to free resveratrol in NCI-H460 cells	[127]
PLGA	EGCG	The EGCG-encapsulated PLGA nanoparticles: - decreased IC_50_ of EGCG from 60 μM (free EGCG) to 9 μM (encapsulated-EGCG) - enhanced the sensitivity of the A549 cells to cisplatin by reducing the dose of cisplatin required by up to 20-fold	[128]

Pterostilbene (*trans*-3,5-dimethoxy-4-hydroxystilbene) is an analogue of resveratrol that occurs naturally and which has similar biological effects [95]. Schneider et al. [96] investigated the effect of pterostilbene (10-100 μM) on two lung cancer cell lines, NCI-H460 and SK-MES-1. Pterostilbene caused a decrease in cell viability, especially for concentrations more than 20 μM, and increased apoptosis and caspase 3 & 7 activity.

## Bioavailability of phenolic compounds

These studies of the biological activity of phenolic compounds against cancer cells shows that there’s great promise for their therapeutic application to treat cancer, but one hindrance to this use is the low absorption they exhibit. The low absorption stems from decreased solubility and decreased stability [97].

Dietary intake of phenolic acids are estimated to range from 200 mg/day up to 1198.6 mg/day [3, 98]. The oral bioavailability of tea catechins seems to be very low, with plasma concentrations being between 5 to 50 times lower than what is required to replicate findings in vitro [99]*.* Another compounding problem is that some phenolic compounds such as quercetin are present in our diets at very low quantities, approximately 20 ± 5 mg of your daily dietary intake [100]. Pharmacokinetics studies has suggested that absorption of quercetin in humans following a single oral dose can be as low as 2% [101]. A study by Hollman et al. [102] showed that when humans received 68 mg quercetin equivalents (nearly twice the estimated maximum flavonol intake) the maximum plasma concentration was only 0.74 μmol/L. Zheng et al. [45] demonstrated that administration of 0.74 μmol/L quercetin caused 5, 10, and 30% inhibition in growth of A549 cells after 24, 48, and 72 h period, respectively. It doesn’t fare much better for diphenylalkaloids such as curcumin either. Many studies have shown that curcumin has a poor oral bioavailability due to low absorption, increased metabolism, and its swift elimination from the body. Yang et al. [103] found the oral bioavailability of curcumin to be 1% when they compared the maximum serum concentrations achieved after intravenous- and oral administration of curcumin, 0.36 ± 0.05 μg/mL and 0.06 ± 0.01 μg/mL, respectively. Another study showed that when 3.6–12 g C complex is taken per day for a week or longer, that the plasma levels of curcumin remains at or below 25 nmol [104]. Siviero et al. [105] reported that after intraperitoneal injection of 100 mg/kg of carbon-14 curcumin, the following distribution was obtained: brain 2.9 ± 0.4 nmol/g, muscle 8.4 ± 6.0 nmol/g, heart 9.1 ± 1.1 nmol/g, lung 16 ± 3 nmol/g, liver 73 ± 20 nmol/g, kidney 78 ± 3 nmol/g, and intestinal mucosa 200 ± 23 nmol/g. This indicates that the bulk of curcumin goes towards the tissue of the intestine.

Resveratrol on the other hand has relatively high oral absorption (at least 70% absorbed), but has a low oral bioavailability [106]. It is postulated that the low oral bioavailability could be due to the rapid sulfate conjugation of resveratrol by the liver/intestine [106]. Several studies concluded that after oral administration of approximately 25 mg resveratrol, the plasma concentration of the free form of resveratrol was between 1 and 5 ng/ml [107–110].

The in vitro studies show what the phenolic compounds can do once accumulated at the site of action and in sufficient concentration. However, in reality concerning oral ingestion, such as when you are trying to get the phenolic compounds from your diet. The phenolic compounds have to navigate many obstacles to get to the bloodstream and ultimately the site of action. The phenolic compounds face the high acidity environment of the stomach which can cause degradation and uncontrolled release which, in turn, can cause decreased absorption from the intestines [97]. Several great reviews have been written on the topic of bioavailability of phenolic compounds, which can aid the reader with further information [111, 112]. Therefore, it is not possible to obtain, through the diet alone, the concentration of phenolic compounds necessary to have the effects described in the previous section. This is one of the questions proposed by Rasouli et al., whether it is possible to achieve the concentration of phenolic compounds in vivo in order to replicate the effects seen in vitro [113]. It is the purpose of the next section to propose that by transporting the phenolic compounds in a way that helps successfully traverse and protect it from these obstacles, it may be possible to achieve similar effects without needing phenolic compounds in the micromolar range.

## Drug delivery

### Nanoparticulate delivery systems

Nanoparticles and liposomes are useful strategies to overcome the poor absorption, rapid metabolism, and elimination inherent in most natural products; helping to increase their bioavailability and target specific sites, such as the lung. This section will be looking at studies carried out on delivery systems using polymeric nanoparticles. For a more comprehensive list of delivery systems utilising other nanoparticulate systems, such as liposomes, micelles, dendrimers, etc. refer to the review by Loira-Pastoriza, Todoroff and Vanbever [110].

Polymeric nanoparticles refers to colloidal systems with spherical or irregular shape that either encapsulates or entraps a biologically active substance [114]. Numerous biodegradable polymers, both synthetic and natural, can be utilised to create polymeric nanoparticles, including polycaprolactone (PCL), polylactic acid (PLA), poly (lactic-co-glycolic acid) (PLGA), chitosan and gelatin [115]. The US Food and Drug Administration (FDA) have approved PLA and PLGA for human applications. PLA and PLGA are broken down in an organism into their biodegradable biocompatible monomeric building blocks, lactic and glycolic acid [115]. When PLA and PLGA are administered intravenously, they are normally quickly cleared by the immune system of the host [116]. To combat this and increase the circulation time, nanoparticles are often coated with poly(ethylene glycol) (PEG), a polymer that can aid in evading clearance by the immune system [117]. Chitosan is a natural polycationic linear polysaccharide, that has been shown to be mucoadhesive, non-immunogenic and non-toxic [118]. Gelatin is a protein based biopolymer that’s highly biocompatible and biodegradable with low toxicity and low antigenicity [119]. The advantages of polymeric nanoparticles include controllable physico-chemical properties, high stability, homogenous size distribution, high drug encapsulation, and controllable drug release [120].

Polymeric nanoparticles have been extensively studied for their drug delivery capacity (refer to table 5 for list of studies discussed below). PLGA is, due to FDA approval, the most popular polymer used for nanoparticle delivery. It is safe and highly stable in colloidal suspensions and has been shown to have controlled release properties [121]. PLGA nanoparticles with or without chitosan coating has been shown to be cytocompatible with A549 cells as high as 5 mg/mL [122]. Khalil et al. [123] showed that orally administered PLGA and PLGA-PEG nanoparticles increases drug absorption (increased bioavailability),causes sustained drug release, and increases the half-life of the encapsulated drug. PLGA and PLGA-PEG nanoparticles increased the peak concentration of curcumin by 2.9- and 7.4-fold, increasing the peak concentration of free curcumin from 4.066 ± 0.564 ng/ml to 11.783 ± 0.454 ng/ml and 29.778 ± 4.632 ng/ml for PLGA and PLGA-PEG nanoparticles, respectively [123]. The PLGA and PLGA-PEG nanoparticles also increased the half-life of the curcumin from 1 h to 4 h and 6 h, for free curcumin, PLGA, PLGA-PEG nanoparticles, respectively [123]. The PLGA and PLGA-PEG nanoparticles enhanced the oral bioavailability of curcumin by 15.6- and 55.4-fold, respectively [123]. Teong et al. [124] encapsulated curcumin in polymeric chitosan, gelatin, and hyaluronan nanoparticles with an encapsulation efficiency of 81, 67, and 78%, respectively. The curcumin-encapsulated- chitosan, gelatin, and hyaluronan nanoparticles all showed enhanced apoptotic effects of 45, 40 and 32%, respectively, as opposed to pure curcumin (>20%) on A549 cells [124]. When administered intravenously to rats, a significant amount of curcumin encapsulated into PLGA nanoparticles were found in the lungs [125]. Kumar et al. [126] used in vitro studies to investigate the effects of naringenin encapsulated chitosan nanoparticles (NAR/CS NPs) on A549 lung cancer cells and normal mouse fibroblast cells (3T3). The results showed that the NAR/CS NPs caused a statistically significant dose-dependent decrease in cell viability in A549 cells as compared with free naringenin, without affecting the normal 3T3 cells [126]. Resveratrol encapsulated in gelatin nanoparticles was shown to induce cell death in human lung cancer cells NCI-H460 by changing the expression of TP53, p21, caspase-3, Bax, Bcl-2 and NF-κB [119]. Previously, it was shown that resveratrol encapsulated gelatin nanoparticles (R-GNPs) had an improved cellular uptake and superior bioavailability, decreasing cell viability, mitochondrial membrane potential and increasing cytotoxicity, DNA damage and intracellular ROS levels as compared to free resveratrol in NCI-H460 cells [127]. Singh et al. [128] encapsulated EGCG in PLGA nanoparticles and assessed it on human lung cancer A549 cells. The EGCG-encapsulated PLGA nanoparticles showed an IC_50_ of 9 μM while the free EGCG showed an IC_50_ of 60 μM, meaning that the nanoparticles reduced the dose required to exert the same antiproliferative effect on the A549 cells by over 6 times [128]. The EGCG encapsulated PLGA nanoparticles also enhanced the sensitivity of the A549 cells to cisplatin by reducing the dose of cisplatin required by up to 20 fold [128]. Phenolic compounds delivered concomitantly with well-established chemotherapeutic drugs were shown to have a synergistic effect. Duan et al. [129] showed that the combined delivery of co-encapsulated curcumin and doxorubicin in poly(butyl cyanoacrylate) nanoparticles reversed the multidrug resistance of the breast cancer cell line (MC7) at a higher efficacy than the agents on its own or in separate nanoparticles. Another study showed that curcumin enhanced the apoptotic effect of doxorubicin while also supressing the adverse effects associated with it [130].

Popov et al. [131] found that administration via intratracheal instillation of fluticasone propionate (FP) encapsulated in poly(lactide)-based mucus-penetrating particles (MPP) for pulmonary delivery showed a higher local exposure to the lungs of rodents as compared to poly(lactide)-based mucoadhesive particles (MAP) and free-FP. PLGA nanoparticles coated with glycol chitosan was shown to be more readily absorbed onto A549 cells than the non-coated PLGA nanoparticles. The chitosan-coated PLGA nanoparticles were found in the lungs up to 72 h after pulmonary administration, whereas non-coated PLGA nanoparticles were removed from the lungs 8 h after administration [132].

### Pulmonary delivery

Pulmonary drug delivery allows for the non-invasive administration of a drug/bioactive compound via inhalation [133]. There are many advantages to delivering drugs via the lungs for both local and systemic treatment, including high bioavailability since the first pass metabolism is bypassed, rapid onset of action due to direct targeting at the site needed (lung cancer cells), self-administration (similar to how asthmatics use their inhalation devices) and non-invasiveness (which increases patient compliance) [134–136]. One of the biggest challenges for cancer chemotherapy is the non-specific targeting/distribution of the anticancer agent and the severe side effects this produces [137]. Nanoparticle-mediated pulmonary delivery will aid in overcoming this obstacle through targeted delivery; reducing the dosage required to treat the cancer and reducing the amount of drug the healthy cells are being exposed to. However, spray-dried nanoparticles are incapable of depositing directly into the lungs since they get exhaled without settling in the alveoli due to their small size range (<1 μm) [138]. The ideal range for particles to be able to deposit in the lung is between 1 and 5 μm [139]. Nanoparticles can be made into the appropriate size via spray-drying the nanoparticles using excipients, such as leucine, to form microparticles. These microparticles can be delivered to the lungs through dry powder inhalers (DPIs). DPIs are portable solid powder delivery devices that are used without the aid of propellants [133]. DPIs often give a better stability profile for the loaded bioactive compound than aerosols or nebulizers [140].

Several drugs have been studied for both local and systemic pulmonary delivery [141]. Polymeric nanoparticles have been used for the pulmonary delivery of small molecules, genes and proteins/peptides [142–148]. However, studies using polymeric nanoparticle-mediated microparticles for pulmonary delivery of phenolic compounds are a little less ubiquitous in the literature.

Scutellarin, a flavone, was incorporated into polymeric microparticles based on poly(vinyl alcohol) (PVA), polyvinylpyrrolidone (PVP) and sodium hyaluronate [149]. The particles showed a median size of 1.95–2.83 μm, which is applicable for inhalation [149]. The particles were administered via pulmonary route and then assessed for bioavailability [149]. It was found that the pulmonary route caused the bioavailability of scutellarin to be 77%, which was 30 fold higher than the oral route [149]. Studies has already shown that these polymeric nanoparticles can be used in combination with common anticancer drugs, such as cisplatin and doxorubicin, to either enhance their efficacy and/or attenuate their adverse effects. Liu et al. [150] prepared paclitaxel- oleic acid-conjugated chitosan nanoparticles (P-OA-CTS) and quercetin-oleic acid-conjugated chitosan nanoparticles (Q-OA-CTS) and then co-loaded both nanoparticles into microparticles by spray-drying the nanoparticles with hydroxypropyl-β-cyclodextrin, lactose, and mannitol as excipients. The microparticles obtained was shown to be in the ideal range of between 1 and 5 μm with a slow release profile [150]. The study ascertained that intravenous delivery of the microspheres caused more accumulation of the encapsulated drug in the liver and kidney than in the lung, while pulmonary administration lead to a significant majority of the drug depositing in the lungs with minimal amounts accumulating in the other organs [150]. Furthermore, 6 h after pulmonary administration, paclitaxel and quercetin concentration in the lung remained high (206.27 μg/g) with comparatively low distribution in the liver (8.82 μg/g), spleen (6.94 μg/g), kidney (5.01 μg/g) and heart (2.61 μg/g) at the same time. Whereas, 6 h after intravenous delivery the concentration of paclitaxel and quercetin in all organs were ≤ 5 μg/g. It was reported that quercetin helped increase the circulatory time of paclitaxel [150]. Combined, this shows that pulmonary delivery of microparticles not only improved the retention time of the drugs, but also allowed for the accumulation of the drug in the lung with only minimal amount of drug accumulating in other organs. This should lead to lower side-effects.

## Conclusion

Phenolic compounds have huge potential in chemoprevention with a plethora of compounds showing promise in in vitro studies. However, the biggest drawback with using phenolic compounds is their low bioavailability due to several factors including low intrinsic activity, malabsorption, high rate of metabolism, inactivity of metabolic products and/or rapid elimination and clearance from the body [76]. It was shown that when the phenolic compounds were incorporated into polymeric nanoparticles, they enhanced the anticancer effects shown in vitro*.* Despite the ability of the polymeric nanoparticle to deliver the phenolic compounds via oral and intravenous administration, it is only natural to assess pulmonary delivery, especially for lung cancer. This is due to the many advantages that pulmonary delivery has. Although there are only a limited amount studies done on pulmonary delivery of phenolic compounds, they do show quite a lot of promise. It would be interesting to see where this field goes in the next few years.

## Abbreviations

4-ACGC: 4-O-(2″-O-acetyl-6″-O-p-coumaroyl-β-d-glucopyranosyl)-p-coumaric acid
5HHMF: 5-hydroxy-3,6,7,8,3′,4′-hexamethoxyflavone
5HPMF: 5-hydroxy-3,7,8,3′,4′-pentamethoxyflavone
AP-1: activator protein-1
AIF: apoptosis-inducing factor
ARRB2: arrestin beta 2
Bcl-2: B cell lymphoma 2
Bad: Bcl-2-associated death promoter
Bax: Bcl-2-associated X protein
JNK: c-Jun N-terminal kinases
COX-2: cyclooxygenase
POLL: DNA polymerase lambda
H69VP: drug-resistant small-cell lung carcinoma
H69: drug-sensitive small-cell lung carcinoma
DPIs: dry powder inhalers
ER: endoplasmic reticulum
EGFR: epidermal growth factor receptor
EGFR-TKIs: epidermal growth factor receptor tyrosine kinase inhibitors
EGCG: Epigallocatechin 3-gallate
EMT: Epithelial-Mesenchymal Transition
ERK1/2: extracellular signal-regulated kinase 1 and 2
GRP78: glucose-regulated protein 78
GADD 153: growth arrest- and DNA damage-inducible gene 153
GADD 45: growth arrest- and DNA damage-inducible gene 45
A549: human lung adenocarcinoma cell line
SPC-A-1: human lung cancer cell line
NSCLC: human non-small cell lung cancer
iNOS: inducible nitric oxide
IC50: inhibitory concentration 50%
mTOR: mammalian target of rapamycin
MMP-9: matrix metalloproteinase-9
MMP-2: matrix metalloproteinase-2
miRNA: microRNA
MAPK1: mitogen-activated protein kinase 1
MAPK14: mitogen-activated protein kinase 14
MEK: mitogen-activated protein kinase and ERK
MAPK3/6: mitogen-activated protein kinase kinases 3/6
MAPK: mitogen-activated protein kinases
MLK3: mixed lineage protein kinase 3
MDM2: mouse double minute 2
MAP: mucoadhesive particles
MPP: mucus-penetrating particles
MUTYH: MutY DNA Glycosylase
Mcl-1: myeloid cell leukemia-1
NAR/CS NPs: naringenin encapsulated chitosan nanoparticles
H2122, H358, H460, H1650, H1975, and H1993: NSCLC cell lines
NFKB1: nuclear factor kappa b subunit 1
NF-κB: nuclear factor kappa-light-chain-enhancer of activated B cells
P-OA-CTS: paclitaxel- oleic acid-conjugated chitosan nanoparticles
PI3K/Akt: phosphoinositide 3-Kinase/protein kinase B
PLGA: poly (lactic-co-glycolic acid)
PARP: poly(ADP-ribose) polymerase
PEG: poly(ethylene glycol)
PVA: poly(vinyl alcohol)
PVP: polyvinylpyrrolidone
PCL: polycaprolactone
PLA: polylactic acid
PCNA: proliferating cell nuclear antigen
PGE2: prostaglandin E2
PTK2: protein tyrosine kinase 2
Q-OA-CTS: quercetin-oleic acid-conjugated chitosan nanoparticles
ROS: reactive oxygen species
RTK: receptor tyrosine kinases
R-GNPs: resveratrol encapsulated gelatin nanoparticles
STAT5A: signal transducer and activator of transcription 5a
STAT3: signal transducer and activator of transcription protein 3
STATs: signal transducer and activator of transcription proteins
SCLC: small-cell lung carcinoma
TGF-β1: transforming growth factor beta 1
TSA: trichostatin A
TNF: tumour necrosis factor
TNFR-1: tumour necrosis factor receptor-1
TP53: Tumour protein p53
TAM: Tyro3, Axl, MerTK
TKI: tyrosine kinase inhibitors
u-PA: urokinase-type plasminogen activator
FDA: US Food and Drug Administration
VEGF: Vascular Endothelial Growth Factor
VEGFA: vascular endothelial growth factor A
